# Biosynthesis of TiO_2_ nanoparticles by *Caricaceae* (Papaya) shell extracts for antifungal application

**DOI:** 10.1038/s41598-022-19440-w

**Published:** 2022-09-24

**Authors:** Abel Saka, Yohannes Shifera, Leta Tesfaye Jule, Bayissa Badassa, N Nagaprasad, R Shanmugam, L Priyanka Dwarampudi, Venkatesh Seenivasan, Krishnaraj Ramaswamy

**Affiliations:** 1 Department of Physics, College of Natural and Computational Science, Dambi Dollo University, Dambi Dollo, Ethiopia; 2 Department of Forestry, College of Agriculture and Veterinary Medicine, Dambi Dollo University, Dambi Dollo, Ethiopia; 3 Centre for Excellence-Indigenous Knowledge, Innovative Technology Transfer and Entrepreneurship, Dambi Dollo University, Dambi Dollo, Ethiopia; 4 Ministry of Innovation and Technology, Addis Ababa, Ethiopia; 5 Department of Mechanical Engineering, ULTRA College of Engineering and Technology, Madurai, Tamil Nadu 625 104 India; 6 TIFAC, CORE-HD, Department of Pharmacognosy, JSS Academy of Higher Education and Research, JSS College of Pharmacy, Ooty, Nilgiris, Tamil Nadu India; 7 Department of Pharmacognosy, JSS Academy of Higher Education and Research, JSS College of Pharmacy, Ooty, Nilgiris, Tamil Nadu India; 8 Department of Mechanical Engineering, Sri Eshwar College of Engineering, Coimbatore, India; 9 Department of Mechanical Engineering, Dambi Dollo University, Dambi Dollo, Ethiopia

**Keywords:** Biophysics, Plant sciences, Engineering, Materials science, Physics

## Abstract

Titanium dioxide nanoparticles (TiO_2_ NPs) were prepared by *Caricaceae* (Papaya) Shell extracts. The Nanoparticles were analyzed by UV–Vis spectrums, X-ray diffractions, and energy-dispersive X-rays spectroscopy analyses with a scanning electron microscope. An antifungal study was carried out for TiO_2_ NP in contradiction of S. *sclerotiorums*, R. *necatrixs* and Fusarium classes that verified a sophisticated inhibitions ratio for S. *sclerotiorums* (60.5%). Germs of pea were individually preserved with numerous concentrations of TiO_2_ NPs. An experience of TiO_2_ NPs (20%, 40%, 80% and 100%), as well as mechanisms that instigated momentous alterations in seed germinations, roots interval, shoot lengths, and antioxidant enzymes, were investigated. Associated with controls, the supreme seeds germinations, roots and plant growth were perceived with the treatments of TiO_2_ NPs. Super-oxide dis-mutase and catalase activities increased because of TiO_2_ NPs treatments. This advocates that TiO_2_ Nanoparticles may considerably change antioxidant metabolisms in seed germinations.

## Introduction

Nanotechnologies are utilized in the areas of medicines, chemistry, environments, energies, agronomy, communications as well as consumer possessions^1^. Metallic oxides with Nanostructures have getting significant interest in numerous fields of technologies^2^. Attentiveness in titanium dioxide (TiO_2_), a metallic oxide, has been growing in current times. Titanium dioxide (Titania) is the furthermost hopeful mineral oxide that is broadly being utilized for fabrications of instruments and other applications^3,4^. TiO_2_ is encouraging for application in the light-emitting device (LCD and LED) that operates in the short wavelength array, from blue light to ultra-violet, as well as in photovoltaic solar cells detectors thin films^5^. Additionally, it is broadly utilized for colourant explained fabrications of transistors as well as field-effect transistors, hybrids and QDSCs (quantum dots solar cells), and Nano generators^6–8^. TiO_2_ Nano Structure of numerous surface morphologies, comprising Nanorods; Nano ropes; Nano threads; Nano ranks; Nano girdles; Nano pointers; Nano prisms; Nano pipes; Nano buds; quantum dot; Nanoparticles; Nanofilms, Nanosheets and Nano plates; Nano microspheres; Nano pyramids; and Nano tetra-pods have applied in varies investigations^9–23^.

Many investigators have conveyed the influences of Titania Nanoparticles on plant germinations as well as development. Titanium as a valuable element rises and helps growth^24^, increases plants productivity by 10–20%^25^ and bio-mass as well as the growth of different plants class^26^ and productions of free radical in propagated seed^27^. The unpredictable outcomes attained from the application of titanium dioxide nanoparticles can designate the positive as well as negative influences of this matter^28^ Other reports stated that titanium dioxide nanoparticles reserved chlorophyll as well as carotenoid at optimum temperature^29^.

Therefore, fabrications of Titanium dioxide Nanostructures are greatly interested all over the biosphere. Titanium dioxide Nanoparticles have received significant consideration because of their exceptional antibacterial, antifungal, UV-filtering characteristics, extraordinary catalytic as well as photochemical activities^30,31^. Fabrications of titanium dioxide nanoparticles are often luxurious, and methods used in the procedure need high energies^32^. Additionally, poisonous diluters and poisonous chemicals are used in these approaches. The substitute technique to prepare these Nanoparticles is biological synthesis. The green approach of nanoparticles through plant’s extract is presently drawing pronounced deals of attention due to their eco-friendly and financial dispensation, scalable, pure surfaces in chemical and greatest considerably their utilization in biology as well as medicines. Several intra-cellular and extra-cellular biological extracts (bacterial, yeasts, fungal, algae and plant) were investigated for the biological synthesis of Nanoparticles and stated their properties such as size shapes compositions chemically towards stability in a medium^33–40^. Biological technologies are used in Bio-synthesis, like usages of plant extracts; it could be a favourite to other techniques. Amongst the biologically objects stated above, plant or their extract appear to be the paramount proxies due to their simply available, appropriate for masses productions of Nanoparticles and wastes product is environmentally friendly dissimilar some micro-organismal extract^41,42^. Phyto constituents in plant extract can concurrently purpose as dropping agents because of the kindly and multipurpose function^43,44^. TiO_2_ Nanoparticles enter the eco-system with incorrect disposals of manufacturing wastes and prevent seeds germinations, seedling development and plant growth.

**Figure 1:**
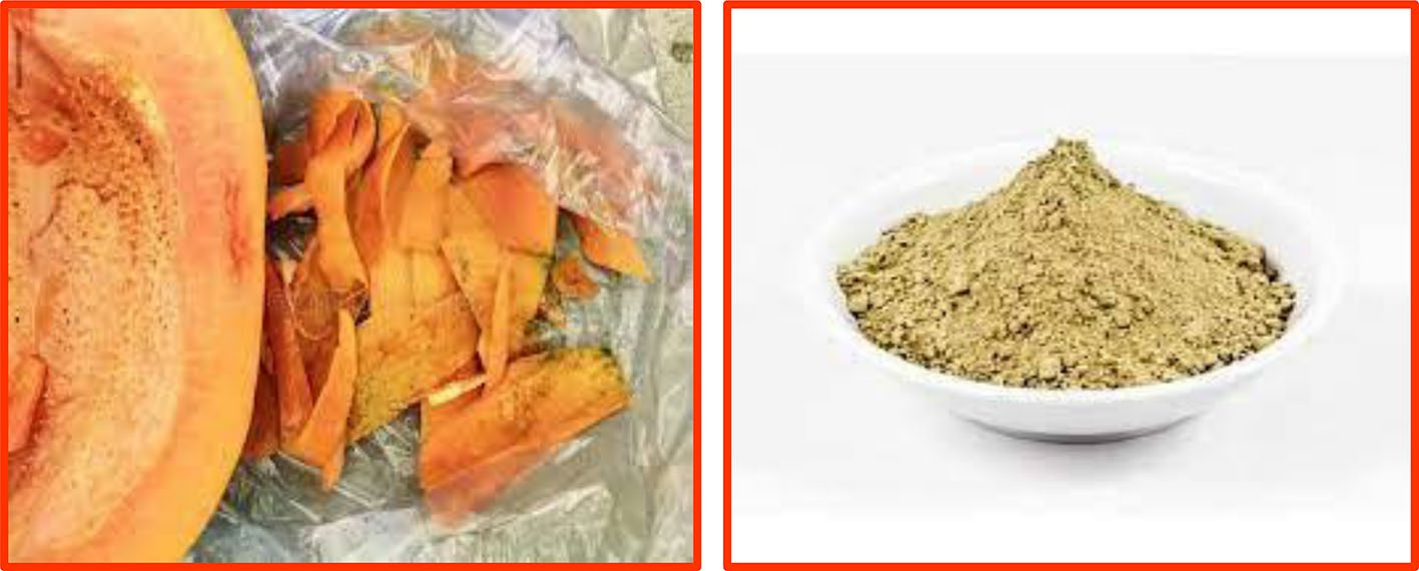
Carica shell preparation.

Several techniques can be useful to avoid these victims. Though, these techniques also have various restrictions on the environment as well as humankind fitness. The utility of Nanoparticles in pathogens controls is recognized as an eco-friendly as well as cost-effective substitute^45^. Nanoparticles are extremely significant in the treatments of plants^46–49^. Carica papaya fits into the family of *Caricaceae* and is usually utilized in treatment as well as control worldwide, particularly in a humid and sub-tropical part of the biosphere. Diverse portions of Carica papaya, like leaves, bark, root, latexes, fruits, flowers, as well as seeds, were used in societies medicals to pleasure diversities of infections^50–53^. Comprising different significant ingredients like vitamins, vitamins (A, E and C) that are a gorgeous basis of antioxidants as well as a mineral-like magnesium (Mg) and potassium (K), vitamin B pantothenic acid as well as foliate and fibres^54^. In the present research, bio-synthesis and analyses of TiO_2_ Nanoparticles through Shell extracts of *Carica papaya* L. and its antifungal activities and seeds germinations were investigated. Since ten years ago, research in the biological synthesis of metallic Nanoparticles through plant extract has released novel views in the area of Nanomedicines^55^. Carica papayas are widespread through the biosphere as well as yield fruits obtainable in every period (Fig. 1). In the present research, Titanium dioxide Nanoparticle was prepared through leafs extracts of Carica papayas in cleans as well as bio-Synthesization technique.

## Materials and methods

### Plants preparations

Titanium Iso-propoxide was bought from Merck Chemicals Ltd, Ethiopia. Carica papaya Shell was peeled and washed. They were scratched into small bits as well as dehydrated at 50 °C. Twenty grams of dry Carica papaya Shells were heated in sanitized water for 30 min. The extracts gained were cleaned via What-man paper Number one and kept in a fridge for more utilization. The preparation of *Carica* papaya Shell extracts is as Fig. 1. The plant we have used in this report was cultivated in the local area of Dambi Dollo Town, Oromia, Ethiopia. This study complies with relevant international, national, institutional and legislative guidelines.

### Biosynthesis of TiO_2_ nanoparticles

A 65 mL 0.2 M titanium Iso-propoxide (99.98%) was equipped in triple distilled water. 15 mL of Carica papaya Shell extracts were gradually mixed dropwise to the solutions at 85 °C with a magnetic stirrer for 5 h, attuned to pH value 11. The occasioning mixtures were centrifuged at 15,000 rpm for 15 min. Pills were splashed as well as centrifuged at 4000 rpm for 15 min. The cleaned pills attained after centrifugations were dehydrated at 55 °C for 5 h and calcite in a soft oven at 455 °C to prepare TiO_2_ Nanoparticles^56^.

### Physical characterization of TiO_2_ nanoparticles

The bio-synthesized Titanium dioxide Nanoparticles were analyzed through the next procedures. Extreme absorbances of the sample were examined through the usage of UV–Visible Spectrophotometry. The physical characterizations of the optical characteristics of titanium oxide nano particle were carried out through ultraviolets and visible absorptions spectroscopy (spectro-photo-meter, Cary-E500 in the ranges of 250 nm–800 nm. X-Ray Diffractions (XRDs) analyses of powders of TiO_2_ nanoparticles were conducted PANalytical X-ray diffractometer functioned at 40 k-V with a current of 30 m-A under Cu-Ka radiations of 2$\theta$ range between 10–80$^\circ$. Dynamics light scatterings (DLSs) were accomplished with Dyna-Pro Plate Readers (Wyatt-Technology). The prepared output was analyzed through transmission electron microscopy [(TEM) Tecnai G2-200 kV with microanalysis]. Scanning-Electron-Microscope (SEM) micrograph was verified through JEOL-JSM-6390 systems as well as elemental plotting was using a similar instrument.

### Preparations of fungal

Platters Potatoes Dextrose Agars (PDAs) Petri plates were subculture for Sclerotinias *sclerotiorums*, Rossellini’s *necatrixs* and *Fusariums* *spp*. Separately, the fungal mycelium tads were provided by the Molecular Plants Microbes Interaction Laboratories. A fungal mycelia tad, scratch through a steriles dagger blades were inoculated at centres of each coagulated steriled PDAs Petri plates that were protected at 20 °C until the fungi matured over an entire surface. The platters were then kept in the fridge at 4 °C for supplementary experimentation uses after being wrapped by Para films.

### Preparations of fungal deferments

Liquefied Culture of three (3) fungal strains-Sclerotinia sclerotium, *Rosellinias* *necatrixs* and Fusarium spp. were equipped through potatoes dextroses broths (PDBs) Mediums, for that fungal mycelium tads were scratch from master platters by using sterilized penknife blades. Each PDBs test tube was protected with 15 mL sterilized PDBs and fungus mycelium bits. This was protected at 20 °C in incubator shakers at 170 rpm for 4–6 days until an adequate development of the fungus mycelium. The test tube was then kept in the fridge at 4 °C. For Spacemen preparations, concentrations of 1 mg/mL of TiO_2_ Nanoparticles were added with 1 mL ultrapure water, in a sterilized eppen-dorf, by energetic shaky for approximately 35 min; after that, the eppen-dorf was centrifuged at 5500 rpm for 15 min. The pills were then wasted, and the supernatants were used for experimentations.

### Determinations of antifungal potentials

The antifungal potentials of syringes filters pasteurized spacemen of TiO_2_ Nanoparticles on *Sclerotinias* *sclerotiorums*, *Rosellinia* *necatrixs* and *Fusariums spp*. were measured in a nearby context^57^. *Sclerotinias* *sclerotiorums*, *Rosellinias* *necatrixs* as well as *Fusariums spp*. were equipped in 15 mL sanitized PDBs, **s**eparately. 2 mL of these suspensions were mixed to each pasteurized yarn persevered test tubes comprising 15 mL of the pasteurized soup mediums to gives finishing volumes of 12 mL. 55 $\upmu$L of TiO_2_ Nanoparticles (needle filters pasteurized) were mixed to define sets of this test-tube for the fortitude of the anti-fungus potentials. A certain identical set of test tubes without a specimen were utilized as a control for the experiments. The test tubes were protected at 20 °C in an incubator shaker at 120 rpm until an adequate development of the fungus mycelium. After 4–6 days, the fungal deferments of wholly test tubes were cleaned usage of What man Filters Papers and weights verified consequently^58,59^.

### Seeds germinations

Seeds feasibility tests were conducted by the floatation techniques. The pea (Cicerarietinum) seeds attained from local markets were laid in beakers of water as was allowable to stand for 6–9 min. Seeds that descended were deliberated variables. Approximately 55 seeds of peas were superficially pasteurized with 0.1% Mercury chloride (HgCl_2_) and cleaned exhaustively with double deionized water several ways^60^. Then seeds were saturated in changed TiO_2_ Nanoparticles suspensions (20%, 40%, 80% and 100%) as well as controlled (water treatments) for an hour at incubators (155 rpm) in 55 mL of solutions. After 1 h, the seed was covered in Petridish comprising moistened filter papers. The Petridish were then positioned in development chambers at 37 °C under a 4: 2 h light: dark photo-period for 12 days. Each petri-dish 10seeds was protected. After the incubations of 12 days, the plantlets germination percentages, roots sizes and shoots sizes were determined for all spacemen^61^.

### Enzymes extractions and analyzes

Shoot sample and root of 550 mg Cicerarietinum were standardized with 2 mL (0.2)M sodium phosphates buffers comprising 0.1% polyvinylpyrrolidones and 20 $\upmu$L 0.05 mM phenylmethanes sulfonyl fluorides. These extracts were centrifuged at 15,000 rpm for 10 min at 4 °C$,$ as well as supernatants were utilized to analyze the enzymes.

### Catalases analyze (CAT)

In the present research, catalases analyzes were calculated using the approach of Cakmak and Horst^62^. The reaction mixtures contained 55 $\upmu$L of H_2_O_2_ (0.3%) with 0.1 mL of enzyme extracts, and the final volumes were completed up to 3 mL by mixing 50 mM phosphates buffers (pH value = 7). The decreases in absorbances were taken for 0–2 min at 240 nm. The CATs activities were communicated as nmol min^−1^ g^−1^ of proteins.

## Result and discussions

**Figure 2:**
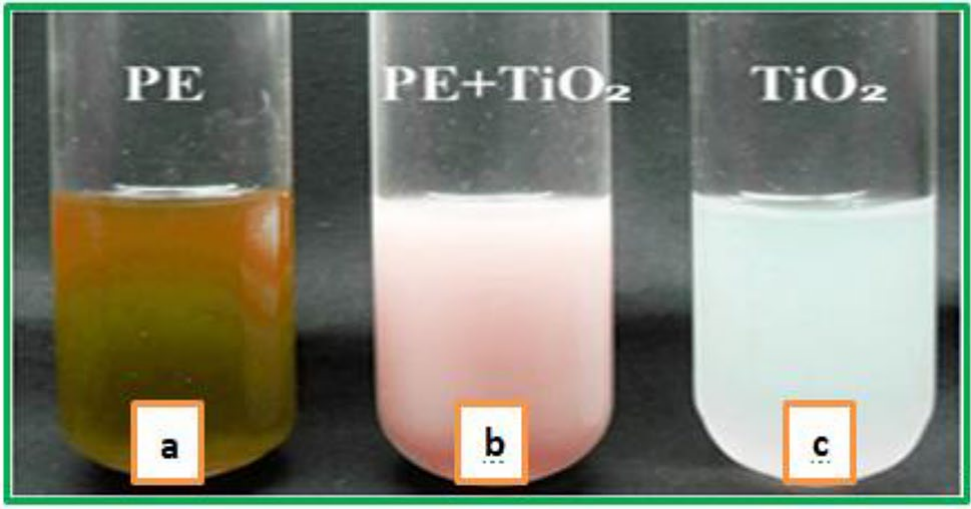
Graphic observations of TiO_2_ nanoparticle preparation (**a**) Titanium Iso-propoxide solution, (**b**) *caricca* papaya Shell extracts, (**c**) changed color.

The prepared TiO_2_ Nanoparticles through green synthesis show colour change, as shown in Fig. 2.

### Analysis of TiO_2_ nanoparticles

**Figure 3:**
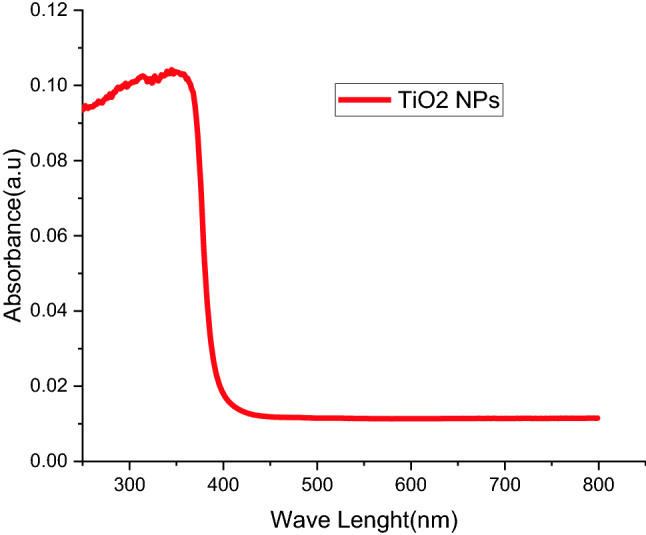
UV-spectra of TiO_2_ nanoparticle from *Carica *papaya shell extracts.

The captivation spectrums of bio-synthesized TiO_2_ nanoparticles by Carica papaya Shell extracts displayed a maximum optical absorptions band at 350 nm (Fig. 3). This absorptions peaks attained were the same as earlier reports. According to the absorption, edges frequently shifted to inferior wavelength or higher energies with declining sizes of nanoparticle^63^.

**Figure 4:**
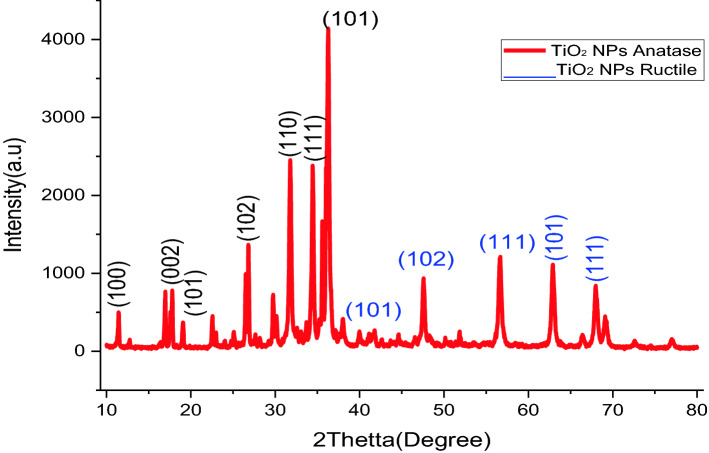
X-ray diffraction patterns of TiO_2_ Nanoparticles from *Carica* papaya shell extracts.

The phenolics groups prohibited agglomerations so that they can be forms metallic Nanoparticles to steady the environment. This advocates that biological molecule is bi-functional in the formations as well as steadying of TiO_2_ nanoparticles in an aqueous intermediate^64^. The X-ray diffraction patterns of biosynthesized TiO_2_ nanoparticles from Shell extracts of C. papaya are presented in (Fig. 4). The separate diffractions peak at 2$\theta$ = 12.76, 18.2, 20.01, 28.34, 32.91, 35.32, 36.57, 40.21, 49.74, 58.34, 64.56 and 70.5 were corresponded to (100), (002), (101), (102), (101), (102),(110),(111), (102), (111), (101), and (111) crystal planes separately. Wholly the deflection peaks were finely indexed to hexagonal phases of both anatase and ructile of TiO_2_. The deflection patterns corresponded to the standards jointly committees on powders diffractions standard (JCPDS) No. 80 to 0075. The X-ray diffraction peaks with great strength disguised that Nanoparticles were greatly crystallized.

The average crystal sizes of biosynthesized spacemen were deliberate by Debye Scherer’s formulas, that is

1 $$ D = \frac{{0.9{\varvec{\lambda}}}}{{\beta c{\text{os}}\theta }} $$

*D* is crystal size, *λ* stands for the length of wave (0.154 nm), *β* is FWHM (full width at half maximums), as well as *θ* (Theta) stands for Bragg diffraction angles. The average crystal size of TiO_2_ nanoparticles exists to be 15 nm^65^. X-ray diffraction patterns gained by present research are identical to the X-R-D pattern attained for previously stated TiO_2_ nanoparticles preparations.

**Figure 5:**
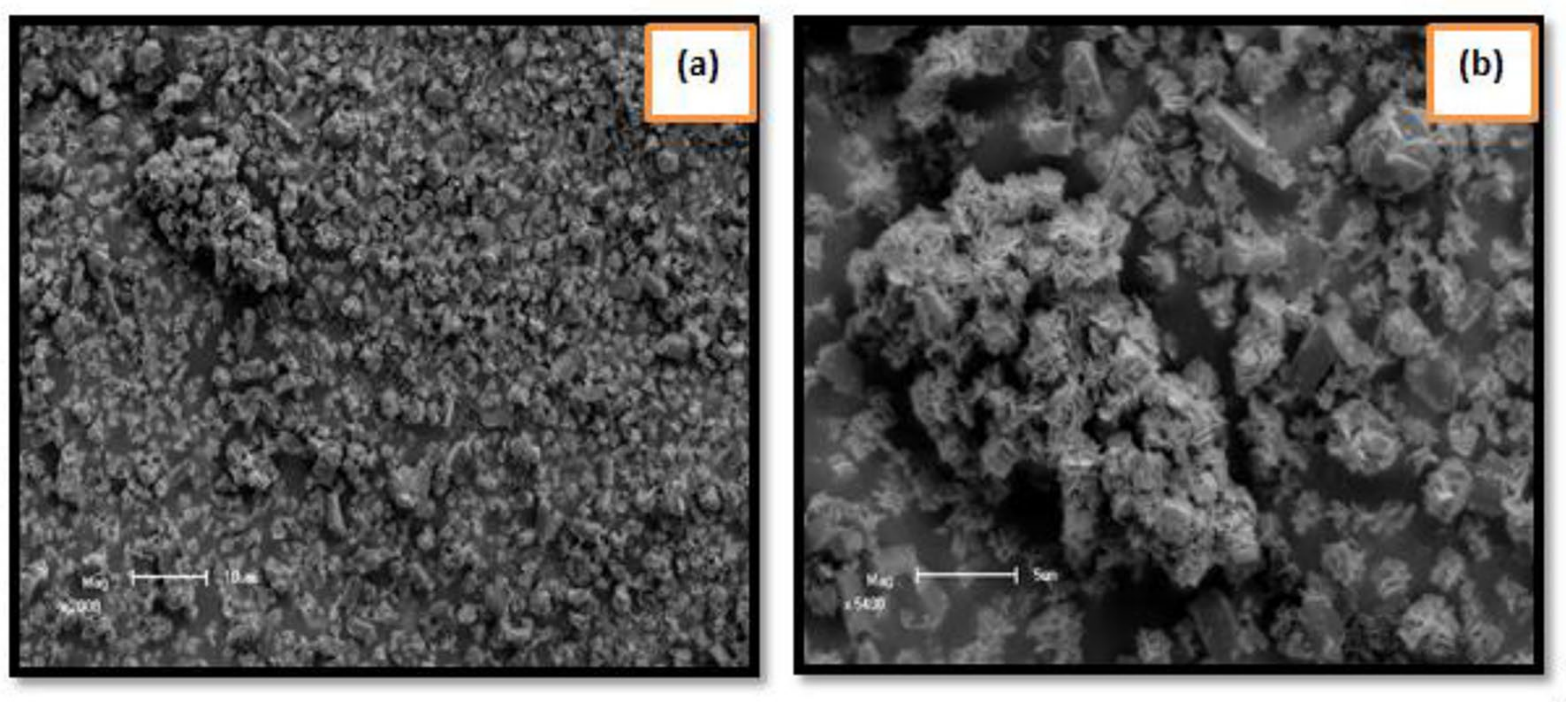
Scanning electron microscope (SEM) images of TiO_2_ nanoparticles from *Carica* papaya shell extracts.

The morphologies of biosynthesized nanoparticles were assessed through scanning electron microscopy (SEM). Figure 5a,b display the superficial morphologies of TiO_2_ Nanoparticles under varied magnification. The scanning electron microscopy (SEM) image shows the agglomeration of separate TiO_2_ nanoparticles. The accumulated images display that convinced particles are semispherical (Fig. 5a) and some monoclinic spherical (Fig. 5b). The formations of floret-like morphologies of TiO_2_ nanoparticles with petals like Nano-sheets can be perceived^66,67^.

**Figure 6:**
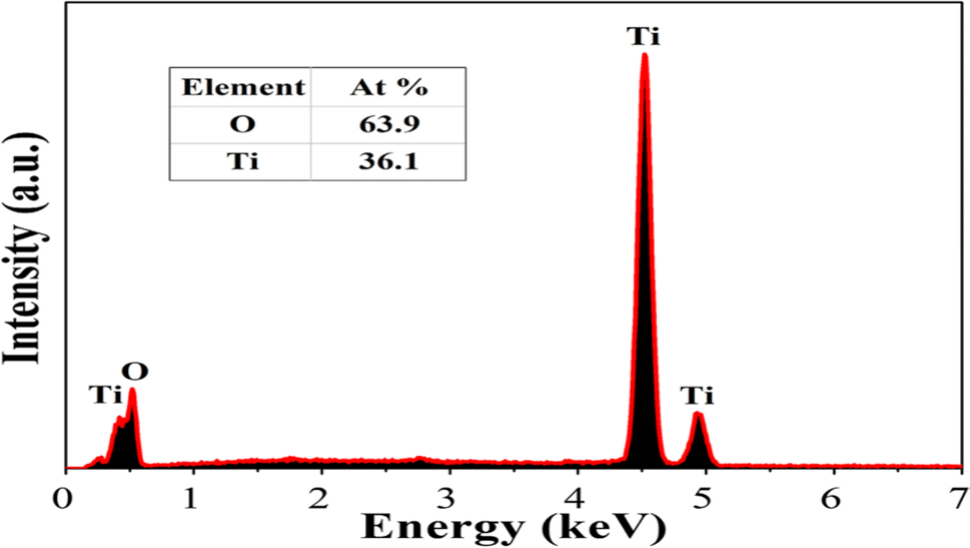
EDX analyzes TiO_2_ nanoparticle biosynthesized from *Carica* papaya shell extracts.

An energy dispersive x-ray diffractive (E-D-X) analysis was conducted for biosynthesized TiO_2_ nanoparticles to identify elementals compositions. A dispersive energy spectrum of sample attained from the scanning electron microscope (SEM) to energy dispersive X-ray diffractive (EDX) analysis displays that samples synthesized by the route have pure TiO_2_ anatase and ructile phases^68–70^. An energy dispersive x-ray diffractive (EDX) approves the existence of Ti and oxygen indications of Titanium dioxide Nanoparticles as displayed in Fig. 6, and its analyses displayed peaks that correspond to optical absorptions of prepared Nanoparticle. The basis of these elements deceits in the bio-components, habitually algae towards TiO_2_ Nanoparticles. Elementals analysis of nanoparticle produced 63.9% of Ti and 36.1% of oxygen (O_2_), which shows the synthesized Nano particle is in its maximum decontaminated forms. The energy dispersive X-ray diffractive (E-D-X) analyses in this research show identical outcomes to previous reports, elemental analyzes of the Nanoparticle produced 36.1% of Titanium and of oxygen 63.9%, respectively^71–73^.

**Figure 7:**
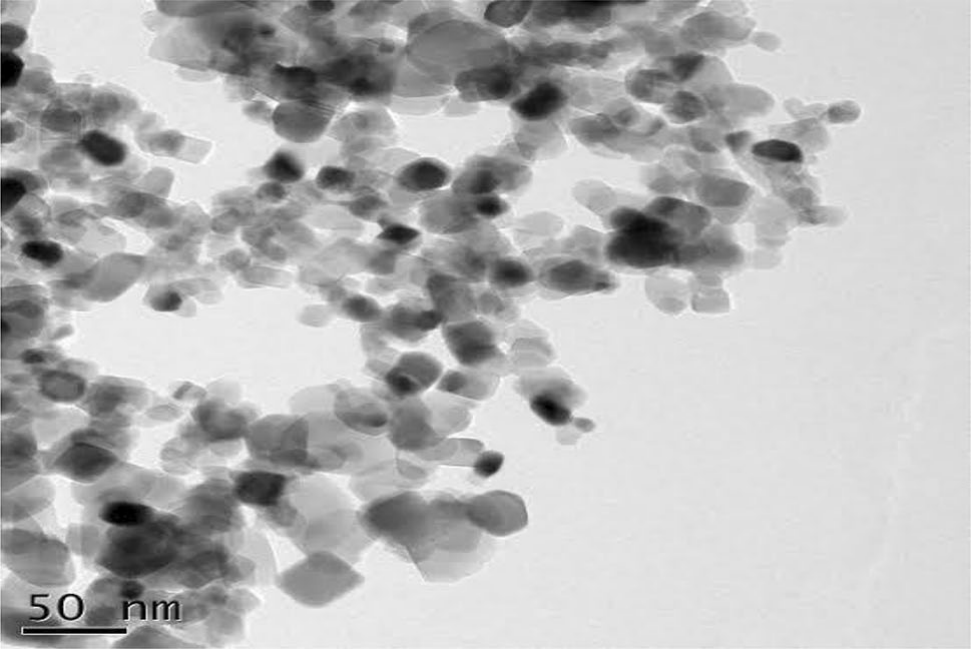
TEM analysis of TiO_2_ nanoparticles biosynthesized from *Carica* papaya shell extracts.

Transmissions electron microscopes (TEMs) depend on the imaging of high energies electron that is passed via a very thin sample. The images acted by the interaction of an electron with a prepared sample are inflamed and absorbed on sensors such as fluorescences screens and photographic film layers cameras. Bisynthesized TiO_2_ nanoparticles were strong-minded in a JEOL 1220 JEM brands transmissions electron microscopes^74^. Transmissions electron microscopes (TEMs) have been utilized for additional studies on the particle sizes, crystal and morphologies of the sample. Transmissions electron microscopes (TEMs) black spherical images of TiO_2_ nanoparticle micro-powders in rutile and anatase phase are given in Fig. 7^75–77^.

### Antifungal activities

Phyto pathogens ground an excessive reduction in crop yields. Fungicide might be the solution for these, but over time, problems of resistance occur^78–81^. Nanoparticles have just the focus of attention with their special antimicrobial effects.

**Figure 8:**
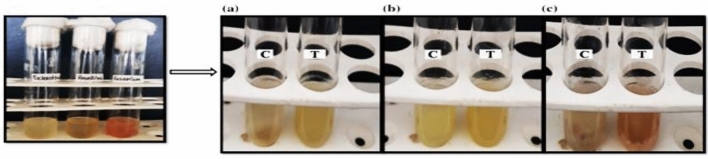
PDBs test-tube for anti-fungus potentials of TiO2 nanoparticles (**a**) *Sclerotinias sclerotiorums*, (**b**) *Rosellinia necatrixs*, (**c**) *Fusariums strains.*

**Figure 9:**
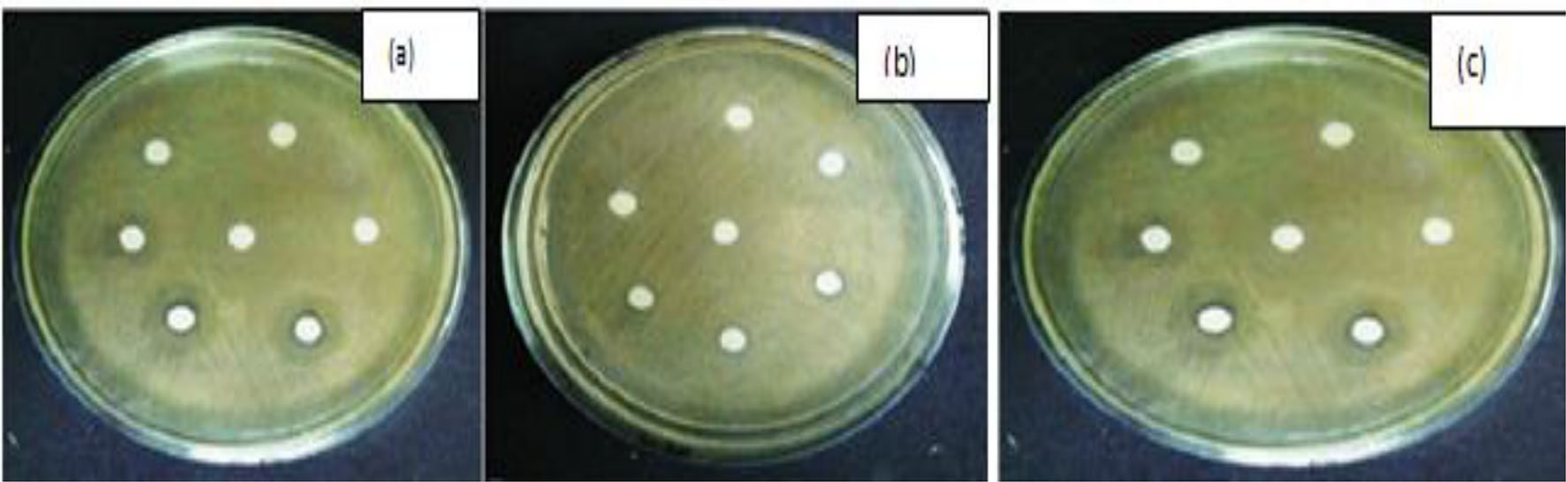
(**a**) *Sclerotinias sclerotiorums*, (**b**) *Rosellinia necatrixs*, (**c**) *Fusariums strains.*

The sets of experimentations for the antifungals potentials purposes of TiO_2_ nanoparticle on *Sclerotinias sclerotium*, *Rosellinia* *necatrixs*, as well as *Fusariums spp*. Revealed fungal mycelial development inhibitions to certain extents in the test tube that was protected with TiO_2_ nanoparticles, as associated with the controls test tube^82^. The outcome was verified after comparisons of the dehydrated weights of the fungal that were on the test-tubes, with as well as without TiO_2_ nanoparticles, consequently, which advocated that TiO_2_ nanoparticles show antifungals activities on the three fungal strains, such that weights (in grams) of parched fungal were bigger for the controls in each case, while associated to test-tube. The comparatives outcomes described graphically (Figs. 8, 9) evidently show the fungal myceliums developments inhibition to some amount in the tests tubes that were immunized with extracts as associated with the controls tests tube.

Nanoparticles could be utilized as a potential anti-fungus agent and help overcome hurdles in fungal disease management modelled by the growth of resistances to conservative fungicide, but various from other where there were little sizes influence, like for PcO6s, *Caenorhabditis-elegans*, and soils bacterial community^83^. Due to the furthermost of the Nanoparticles accumulated in liquefied broths, it is pretty possible that supplementary modifications of the metal oxide nanoparticles in mediums may have happened after additions of agars to coagulate the mediums.

### Seeds germinations

**Figure 10:**
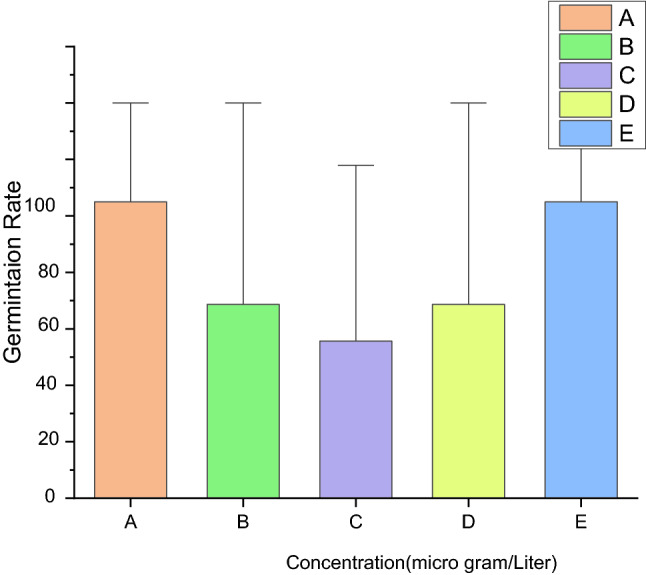
Germinations of antifungals potential (**A**) Controls (**B**) TiO_2_ 20%, (**C**) TiO_2_ 40%, (**D**) TiO_2_ 80% (**E**)TiO_2_ 100%.

Seed germinations are speedily increasing procedure and broadly utilized for phytotoxicity analyses, and also have the advantage of sensitivities, simplicities, cost-effective as well as appropriateness for verified chemicals specimens^84^. In the present research, the influences of TiO_2_ nanoparticles on germinations of peas were studied. Figure 10 displays the influence of TiO_2_ nanoparticles biosynthesized from Caricas papaya shell extracts on pea germinations over 12 days. All concentrations of TiO_2_ nanoparticles increased shoots as well as roost elongated. In these experiments, the lengths of the roots after 12 days increased, owing to bigger absorptions. Figure 10 showed that a concentration of TiO_2_ NP 100% observed has a higher performance of roots lengths. The growths in roots and shoot lengths have gotten supreme with TiO_2_ nanoparticles (100%). Nevertheless, for the seeds preserved with 20%, 40% and 80% of the TiO_2_ nanoparticles, the standard errors were unimportantly inferior for roots and shoot lengths. In identical research^85^ Titanium oxide nanoparticle was prepared from liquefied extracts of *Elaeagnus-Angustifolia*.

## Conclusion

In the present study, Titanium dioxide nanoparticles (TiO_2_NPs) were biosynthesized by using Carica papaya Shell extract, as well as nanoparticles prepared as well as analyzed. Crystalline average sizes measured 15 nm. In addition, it was observed that TiO_2_ NPs were semispherical as well as mono-clinic not spherical. In antifungal studies was perceived that biosynthesized TiO_2_ nanoparticles displayed antifungal influence in contradiction of *Sclerotinias sclerotiorums Fusariums spp*, as well as *Rosellinia necatrixs*. The effects of TiO_2_ NPs on seeds germinations, 100% of TiO_2_ NPs are furthermost appropriate for cultivating the roots as well as shoot lengths. TiO_2_ NPs were biosynthesized through Carica-papaya shell extracts in cheaper, eco-friendly techniques with biological preparation techniques. These biosynthesized TiO_2_ NPs could be utilized to control the reproductions of pathogenic fungus that damages plant growths. The outcomes of this study will give novel comprehensions into the effectiveness of biological methods. Additionally, this research will tile the ways for an optimistic step in the direction of a green strategy for the preparations of metallic oxides nanoparticles and the utilization of their biopotentials in agricultural areas. However, the special properties of various influences like doses, toxicities, real ecological circumstances etc., on germinations and plantlet growths of floras need to be investigated auxiliary in relation to biological methods.

## Data Availability

The datasets used and analyzed during the current study are available from the corresponding author on request.
